# Case Report: Multiple Retinal Astrocytic Hamartomas in Congenital Disorder of Glycosylation-Ia

**DOI:** 10.3389/fmed.2022.697030

**Published:** 2022-02-14

**Authors:** Giulia Midena, Elisabetta Pilotto

**Affiliations:** ^1^IRCCS—Fondazione Bietti, Rome, Italy; ^2^Department of Ophthalmology, University of Padova, Padua, Italy; ^3^ERN-EYE Center, Padova University Hospital, Padua, Italy

**Keywords:** congenital disorder of glycosylation-Ia, astrocytic hamartoma, retinal dystrophy, multimodal imaging, OCT, autofluorescence, metabolic disease

## Abstract

Congenital disorder of glycosylation-Ia (CDG-Ia) is a rare autosomal recessive genetic disorder, characterized by systemic and ophthalmological abnormalities. Here, we report multiple retinal astrocytic hamartomas as a new retinal finding in an adolescent affected by congenital disorder of CDG-Ia. A 15-year-old boy affected by CDG-Ia underwent full ophthalmic examination, full field electroretinography (ERG) evaluation and retinal multimodal imaging, including: fundus photography, spectral domain optical coherence tomography (SD-OCT) and blue fundus autofluorescence (FAF). Blue FAF showed multiple papillary and iuxtapapillary bilateral hyper-FAF lesions, corresponding to hyperreflective thickening of the retinal nerve fiber layer, with internal optical empty spaces and posterior dense optical shadowing at SD-OCT. These imaging findings were consistent with retinal astrocytic hamartomas. Scotopic ERG response was significantly reduced in both eyes. Macular edema and absence of the retinal outer segments layer were also detectable. Retinal multi-modal imaging provides additional insights about retinal involvement of patients affected by CDG-Ia. In particular, this case shows the presence of multiple retinal astrocytic hamartomas.

## Introduction

Congenital disorder of glycosylation (CDG) Ia, also known as phosphomannomutase 2 (PMM2)-CDG, is a rare autosomal recessive genetic disorder, characterized by neurometabolic abnormalities (1–3). Main clinical features are: facial dysmorphism, abnormal fat distribution, various coagulation and endocrine defects. Neurologic, cardiac, gastrointestinal, hepatic, renal, immunologic, and skeletal abnormalities may be also present (1, 2). High myopia, abnormal eye movements, squint, cataract, nystagmus and retinal dystrophy have been reported (3–7).

## Case Report

**Figure 1:**
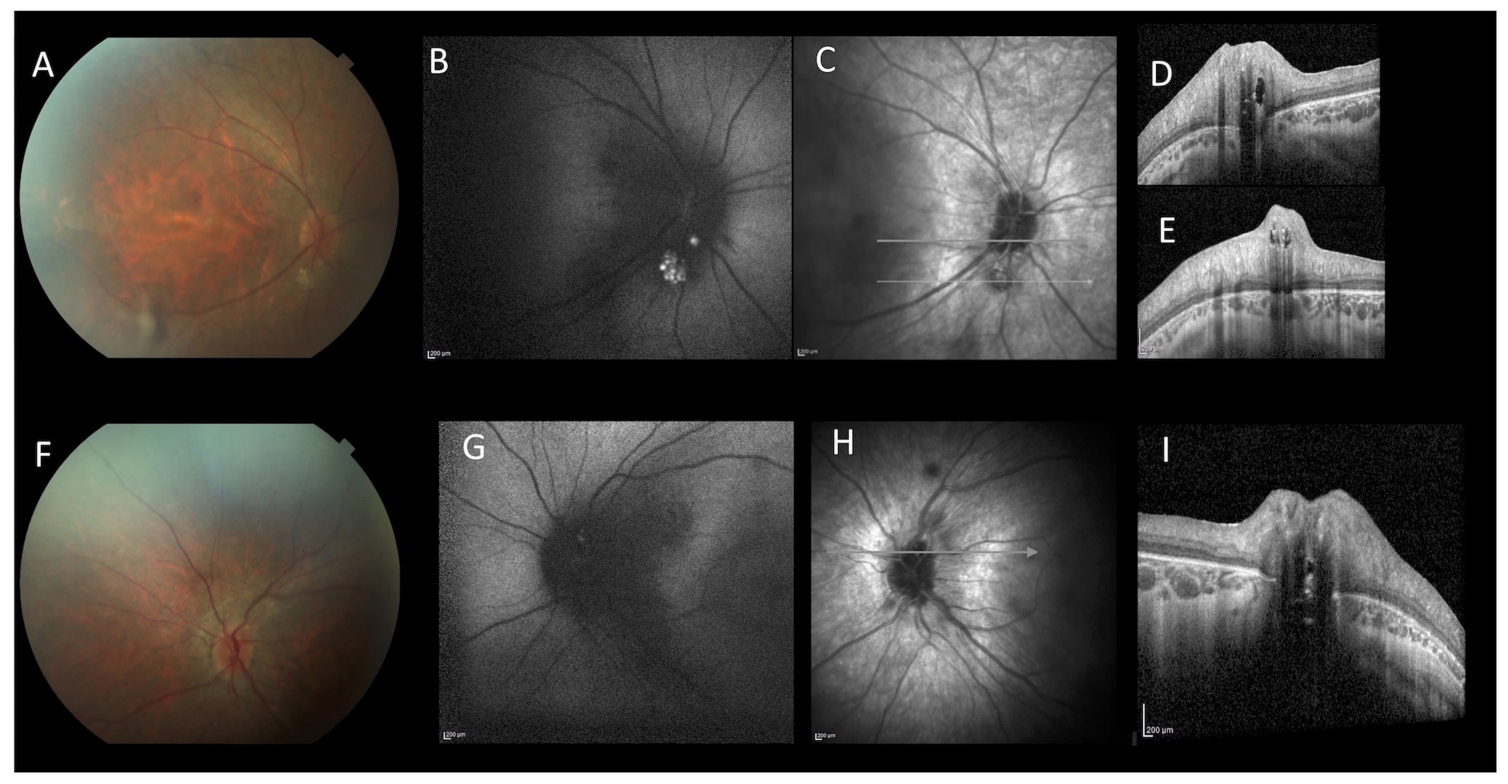
Multimodal imaging of a 15-year-old patient affected by CDG-Ia. Color fundus photograph of the right **(A)** and left **(F)**. Blue-autofluorescence (FAF, **B,G**) shows hyperautofluorescent spots. Linear SD-OCT **(C–E,H,I)** shows hyperreflective thickening of the retinal nerve fiber layer RNFL with internal moth-eaten optical empty spaces and posterior dense optical shadowing. These aspects are diagnostic for multiple, bilateral, calcific retinal astrocytic hamartomas.

**Figure 2:**
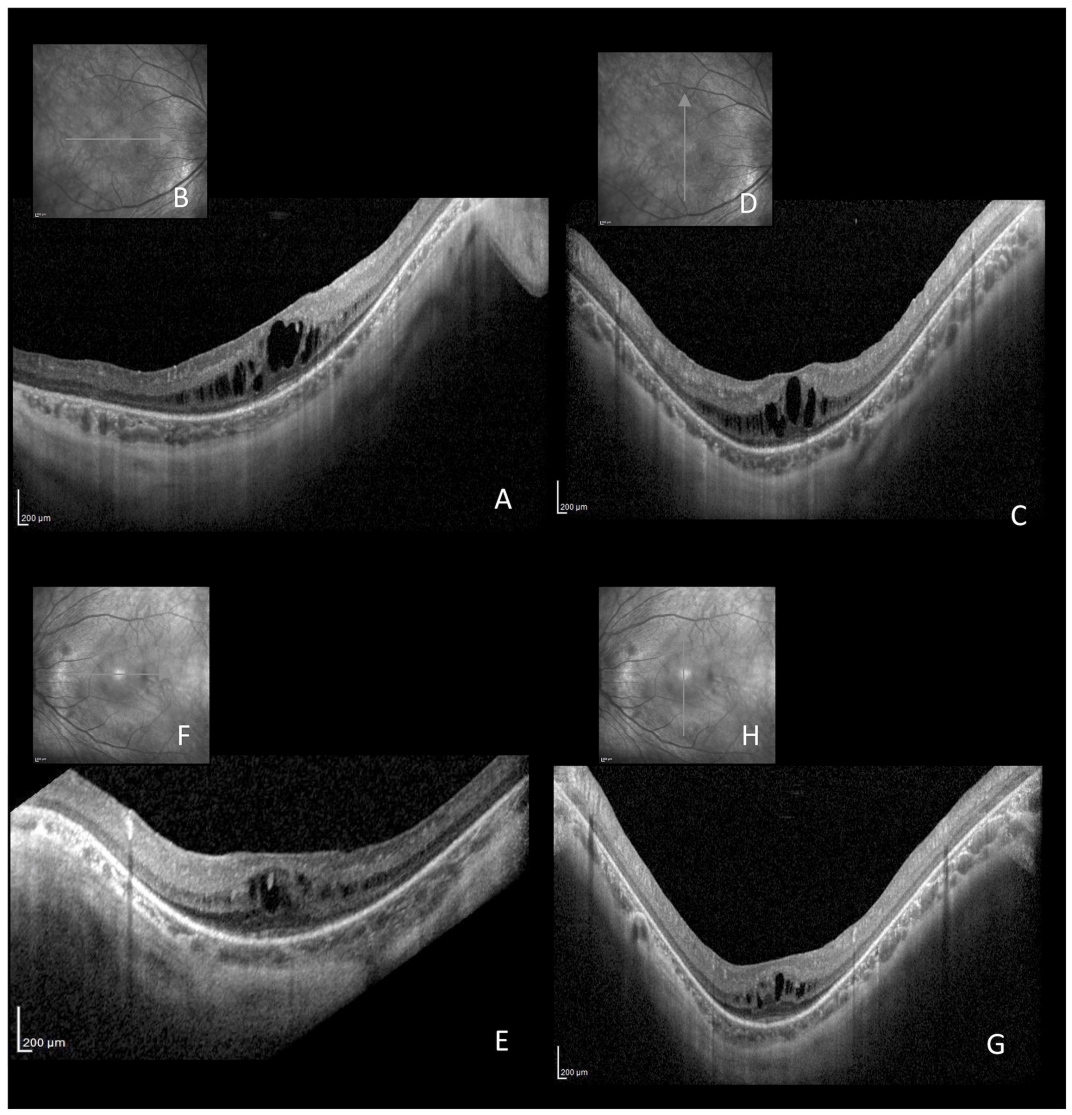
Horizontal **(A,B,E,F)** and vertical **(C,D,G,H)** linear SD-OCT passing through the fovea of the same patient affected by CDG-Ia. Cystoid macular edema with foveal and perifoveal retinal thickening, intraretinal cysts, retinoschisis, and absence of the retinal outer segment, outside the fovea, are evident.

A 15-year-old boy affected by, genetically confirmed, CDG-Ia complaining progressive high myopia, visual acuity impairment and night blindness, was referred to our pediatric low vision unit. His systemic history was characterized by: hypotonia, abnormal fat distribution, joint contracture, developmental delay and feeding difficulties. Eye examination showed: bilateral high myopia (−8.0 diopters), best-corrected visual acuity of 20/40 in both eyes, exotropia and hypertropia. Fundus biomicroscopy of the right eye revealed vitreous disorganization and, only in the right eye, a iuxtapapillary, white, gliotic tissue. Optic disc of the left eye appeared normal. Blue fundus autofluorescence (FAF) of the right eye showed multiple intensely hyper-FAF spots corresponding to the gliotic tissue, and an isolated hyper-FAF spot on the optic nerve head. Blue FAF of the left eye showed a mild hyper-FAF spot on the optic nerve head. Spectral domain optical coherence tomography (SD-OCT) of these lesions showed hyperreflective thickening of the retinal nerve fiber layer (RNFL) with internal moth-eaten optical empty spaces and posterior dense optical shadowing. The combined imaging techniques were diagnostic for multiple, bilateral, calcific retinal astrocytic hamartomas (Figure 1). In particular, according to the SD-OCT findings, we excluded the diagnosis of optic disc drusen. SD-OCT also showed convex scleral profile, foveal and perifoveal retinal thickening with intraretinal cysts, retinoschisis, and the absence of the retinal outer segments, outside the fovea (Figure 2). Full field electroretinography (ERG) evaluation according to ISCEV standards revealed a significant reduction of scotopic response with mild reduction in photopic response in both eyes. Brain magnetic resonance imaging and specific genetic blood tests excluded the diagnosis of tuberous sclerosis and neurofibromatosis type 1, systemic syndromes typically characterized by multiple, bilateral, calcific retinal astrocytic hamartomas.

## Discussion/Conclusion

CDG-Ia is the most common congenital disorder of N-glycosylation. Mutations in the PMM2 gene, located on chromosome 16p13, cause a deficiency of PMM. This cytoplasmic enzyme has an essential role in the N-glycosylation process and in the synthesis of glycosylphosphati-dylinositol, which is used to anchor proteins to the cell membrane (1, 6, 7). Immunohistochemical studies of the mammalian retina have established that N-linked glycans are represented in all retinal layers: this may explain the previously known retinal manifestation of CDG in the human retina, namely retinal dystrophy (8, 9). The peculiarity of this case is the previously unreported combination of CDG-Ia and the presence of multiple, bilateral, calcific retinal astrocytic hamartomas. These benign retinal lesions are glial tumors located in the RNFL, arising from retinal astrocytes (10). Clinically they appear as cream-white, well-circumscribed, non-calcified or calcified, elevated lesions that may present multiple or solitary. These lesions are commonly calcified and multilobulated in appearance, as in our case, but may also present flat and semitraslucent. Multiple and bilateral retinal astrocytic hamartomas are most frequently associated with tuberous sclerosis and neurofibromatosis type 1, and rarely, retinitis pigmentosa (10, 11). In our case, any other phacomatosis were excluded. Retinal astrocytic hamartomas originate from the astrocytes in the RNFL. These cells are characteristic star-shaped glial cells located in the retina, and also in the brain and spinal cord (12). Recent findings revealed that central nervous system astrocytoma progression is correlated with the constant decrease of total N-glycosylation. In particular, Padhiar et al. demonstrated that brain astrocytoma is characterized by the loss of overall N-glycosylation (12). Since brain astrocytoma and retinal astrocytic hamartoma share the same originating cell, we hypothesize that the abnormal proliferation of retinal astrocytes may be due to the deficit of N-glycosylation. Concerning the outer retinal layers, Andreasson et al. hypothesized that in the CDG-Ia retinal disease opsin and interphotoreceptor retinoid-binding protein, the two major glycosylated proteins associated with photoreceptors, are affected (13). Therefore, the lack of PMM determines the inefficacy of these proteins and a progressive photoreceptor degeneration, which may cause, as late phenomenon, a pigmentary retinopathy (5–7). Pigment accumulation is a late stage clinical sign, reported only after significant photoreceptor cell death and migration of the retinal pigment epithelium cells toward the inner layers of the retina (5–7). Jensen et al. rarely noted pigment deposition in a series of 23 pediatric patients with CDG-Ia, but it was described by Krasnewich et al. in his adult patients (7, 13). Marked pigmentary fundus changes were undetectable in our patient, however, both SD-OCT and ERG findings were consistent with rod dystrophy. This might indicate that the patient was young or, probably, CDG-Ia retinal disease was at an early stage, and therefore the diagnosis of retinitis pigmentosa cannot be excluded.

In conclusion, the present case confirms the essential role of retinal multimodal imaging which provides, in a non-invasive way, new insights in the retinal involvement of patients affected by CDG-Ia. In particular, retinal multimodal imaging was instrumental to document the association, never reported before, between CDG-Ia and multiple, bilateral, calcific retinal astrocytic hamartomas. These new findings should be considered in the multidisciplinary evaluation of children within CDG, and retinal and/or brain astrocytic hamartoma, which share the same originating cell, should be excluded.

## Data Availability Statement

The original contributions presented in the study are included in the article/supplementary material, further inquiries can be directed to the corresponding author.

## Ethics Statement

Written informed consent was obtained from the minor(s)' legal guardian/next of kin for the publication of any potentially identifiable images or data included in this article.

## Author Contributions

GM and EP: data research, case design, drafting and revising, final approval, and agreement to be accountable for all aspects of the work.

## Conflict of Interest

The authors declare that the research was conducted in the absence of any commercial or financial relationships that could be construed as a potential conflict of interest.

## Publisher's Note

All claims expressed in this article are solely those of the authors and do not necessarily represent those of their affiliated organizations, or those of the publisher, the editors and the reviewers. Any product that may be evaluated in this article, or claim that may be made by its manufacturer, is not guaranteed or endorsed by the publisher.
